# Effect of pretransplant dialysis vintage on clinical outcomes in deceased donor kidney transplant

**DOI:** 10.1038/s41598-022-20003-2

**Published:** 2022-10-21

**Authors:** Jeong-Hoon Lim, Yena Jeon, Deok Gie Kim, Yeong Hoon Kim, Joong Kyung Kim, Jaeseok Yang, Myoung Soo Kim, Hee-Yeon Jung, Ji-Young Choi, Sun-Hee Park, Chan-Duck Kim, Yong-Lim Kim, Jang-Hee Cho, The Korean Organ Transplantation Registry Study Group, Myoung Soo Kim, Jaeseok Yang, Jin Min Kong, Oh Jung Kwon, Deok Gie Kim, Cheol Woong Jung, Yeong Hoon Kim, Joong Kyung Kim, Chan-Duck Kim, Ji Won Min, Sik Lee, Yeon Ho Park, Jae Berm Park, Jung Hwan Park, Jong-Won Park, Tae Hyun Ban, Sang Heon Song, Seung Hwan Song, Ho Sik Shin, Chul Woo Yang, Hye Eun Yoon, Kang Wook Lee, Sang-Ho Lee, Su Hyung Lee, Yu Ho Lee, Jung Pyo Lee, Jeong-Hoon Lee, Jin Seok Jeon, Heungman Jun, Kyung Hwan Jeong, Ku Yong Chung, Jong Soo Lee, Dong-Wan Chae, Soo Jin Na Choi, Sung Shin, Seungyeup Han, Kyu Ha Huh

**Affiliations:** 1 Department of Internal Medicine, School of Medicine, Kyungpook National University, Kyungpook National University Hospital, 130 Dongdeok-Ro, Jung-Gu, Daegu, 41944 South Korea; 2 Department of Statistics, Kyungpook National University, Daegu, South Korea; 3 Department of Surgery, Yonsei University Wonju College of Medicine, Wonju Severance Christian Hospital, Wonju, South Korea; 4 Department of Internal Medicine, Inje University Busan Paik Hospital, Busan, South Korea; 5 Department of Internal Medicine, Bongseng Memorial Hospital, Busan, South Korea; 6 Department of Internal Medicine, Yonsei University College of Medicine, Seoul, South Korea; 7 Department of Surgery, Yonsei University College of Medicine, Seoul, South Korea; 8 Department of Nephrology, BHS Hanseo Hospital, Pusan, Republic of Korea; 9 Department of Surgery, College of Medicine, Han Yang University, Seoul, Republic of Korea; 10 Department of Surgery, Korea University Anam Hospital, Seoul, Republic of Korea; 11 Division of Nephrology, Department of Internal Medicine, Bucheon St. Mary’s Hospital, Bucheon, Republic of Korea; 12 Department of Internal Medicine, Jeonbuk National University Hospital, Jeonju, Republic of Korea; 13 Department of Surgery, Gil Medical Center, Gachon University College of Medicine, Incheon, Republic of Korea; 14 Department of Surgery, Samsung Medical Center, Sungkyunkwan University School of Medicine, Suwon, Republic of Korea; 15 Department of Nephrology, Konkuk University School of Medicine, Seoul, Republic of Korea; 16 Department of Nephrology, Yeungnam University Hospital, Daegu, Republic of Korea; 17 Division of Nephrology, Department of Internal Medicine, Eunpyeong St. Mary’s Hospital, Seoul, Republic of Korea; 18 Department of Internal Medicine, Pusan National University Hospital, Pusan, Republic of Korea; 19 Department of Surgery, Ewha Womans University Seoul Hospital, Seoul, Republic of Korea; 20 Department of Internal Medicine, Division of Nephrology, Kosin University College of Medicine, Pusan, Republic of Korea; 21 Division of Nephrology, Department of Internal Medicine, Seoul St. Mary’s Hospital, Seoul, Republic of Korea; 22 Department of Internal Medicine, College of Medicine, Incheon St. Mary’s Hospital, The Catholic University of Korea College of Medicine, Bucheon, Republic of Korea; 23 Department of Nephrology, Chungnam National University Hospital, Daejeon, Republic of Korea; 24 Department of Nephrology, Kyung Hee University Hospital at Gangdong, Seoul, Republic of Korea; 25 Department of Surgery, Ajou University School of Medicine, Suwon, Republic of Korea; 26 Division of Nephrology, Department of Internal Medicine, CHA Bundang Medical Center, CHA University, Seongnam, Korea; 27 Department of Nephrology, SMG-SNU Boramae Medical Center, Seoul, Republic of Korea; 28 Department of Surgery, Myongji Hospital, Goyang, Republic of Korea; 29 Department of Internal Medicine, Soonchunhyang University Seoul Hospital, Seoul, Republic of Korea; 30 Department of Surgery, Inje University Ilsan Paik Hospital, Goyang, Republic of Korea; 31 Department of Internal Medicine, Kyung Hee University College of Medicine, Seoul, Republic of Korea; 32 Department of Surgery, Ewha Womans University Mokdong Hospital, Seoul, Republic of Korea; 33 Department of Internal Medicine, Ulsan University Hospital, Ulsan, Republic of Korea; 34 Division of Nephrology, Seoul National University Bundang Hospital, Seoul, Republic of Korea; 35 Department of Surgery, Chonnam National University Medical School, Gwangju, Republic of Korea; 36 Department of Surgery, Asan Medical Center, Seoul, Republic of Korea; 37 Department of Internal Medicine, Keimyung University School of Medicine, Daegu, Republic of Korea

**Keywords:** Nephrology, Outcomes research

## Abstract

The waiting time for deceased donor kidney transplants (DDKT) is increasing. We evaluated DDKT prognosis according to the pretransplant dialysis vintage. A total of 4117 first-time kidney transplant recipients were enrolled from a prospective nationwide cohort in Korea. DDKT recipients were divided into tertiles according to pretransplant dialysis duration. Graft failure, mortality, and composite were compared between DDKT and living donor kidney transplant (LDKT) recipients. Pretransplant dialysis vintage was longer annually in DDKT recipients. In the subdistribution of the hazard model for the competing risk, the first tertile did not show an increased risk of graft failure compared with LDKT recipients; however, the second and third tertile groups had an increased risk of graft failure compared to LDKT recipients (adjusted hazard ratio [aHR] 3.59; 95% confidence interval [CI] 1.69–7.63; *P* < 0.001; aHR 2.37; 95% CI 1.06–5.33; *P* = 0.037). All DDKT groups showed a significantly higher risk of patient death than LDKT, with the highest risk in the third tertile group (aHR 11.12; 95% CI 4.94–25.00; *P* < 0.001). A longer pretransplant dialysis period was associated with a higher risk of the composite of patient death and graft failure in DDKT recipients. DDKT after a short period of dialysis had non-inferior results on graft survival compared with LDKT.

## Introduction

Kidney transplantation (KT) is the best treatment option with many advantages over dialysis in patients with end-stage kidney disease (ESKD). Although deceased donor kidney transplant (DDKT) has a much better prognosis than maintenance dialysis patients^1,2^, it has a worse prognosis than living donor kidney transplant (LDKT). The waiting time for DDKT is steadily increasing because of the growing gap between the rapidly increasing demand for organs and the slowly increasing donated organs^3^.

The pretransplant dialysis duration affects the outcomes after KT. Some differences have been found in the safety window of the dialysis period before KT. The shorter the pretransplant dialysis period, the better the prognosis after KT^4–7^. Our recent study of a Korean LDKT cohort also confirmed that pretransplant dialysis longer than 6 months increases the risk of graft failure^8^. However, most studies that evaluated the association of pretransplant duration and prognosis analyzed LDKT and DDKT recipients without separating them. Few studies have involved DDKT recipients, and no comparison with LDKT has been made^4,9,10^.

Pretransplant dialysis duration might not be a major obstacle due to recent progress in dialysis technology and methods^11–13^. However, many patients with ESKD included in the studies on the association between pretransplant dialysis duration and posttransplant prognosis had received KT in the last century or decades ago, which does not reflect the recent dialysis trend^9,14–16^. In addition, differences among countries exist in the proportion of each dialysis method, preferred dialysis membrane, and detailed dialysis settings such as blood flow rate^11,17^. Different ethnic backgrounds and adherence may also affect the association between dialysis duration and transplant outcome^18,19^.

This study evaluated the effect of pretransplant dialysis vintage on the clinical outcomes of DDKT recipients using a nationwide Korean cohort of KT recipients who had recently received the transplant. It will allow us to ascertain the impact of the waiting period on the prognosis after KT.

## Results

### Baseline characteristics

**Figure 1:**
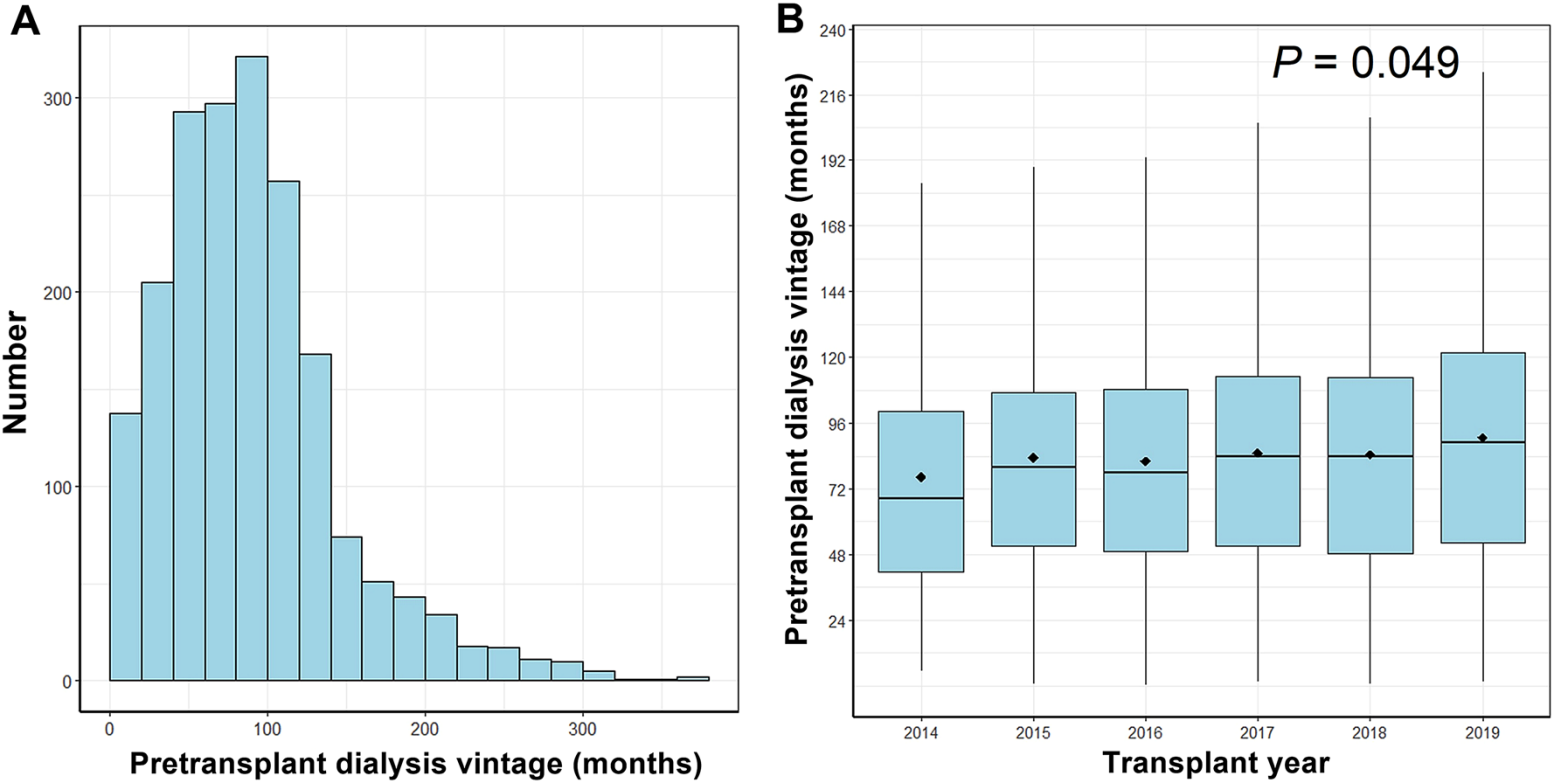
Pretransplant dialysis vintage distribution and annual changes in deceased donor kidney transplant. *LDKT* living donor kidney transplant.

The distribution and annual change in pretransplant dialysis vintage are shown in Fig. 1. The median pretransplant dialysis period was 83.1 months (interquartile range [IQR] 50.4–114.8 months) in DDKT recipients. The trend analysis shows the pretransplant dialysis duration gradually increased every year (*P* = 0.049).

A comparison of baseline characteristics between LDKT and DDKT patients stratified by pretransplant dialysis vintage is shown in Table 1. The first tertile was 648 patients with pretreatment dialysis for up to 60.5 months (median 38.3 months), the second tertile was 651 patients between 60.5 and 101.8 months (median 83.1 months), and the third tertile was 647 patients over 101.8 months (median 131.7 months). The DDKT patients were older on average than the LDKT patients (*P* < 0.001). The proportion of patients with pretransplant desensitization was almost one-third of the LDKT group but was rare in the DDKT groups (*P* < 0.001). Deceased donors had diabetes and hypertension more frequently compared with living donors (both *P* < 0.001). The proportion of induction immunosuppressant of anti-thymocyte globulin was higher in DDKT recipients than in LDKT recipients (*P* < 0.001), and it was the highest in the third tertile group (33.0%). The proportion of maintenance use of tacrolimus was also higher in DDKT recipients (*P* < 0.001).

**Table 1:** Baseline characteristics.

	LDKT (n = 2171)	Tertile 1 (n = 648)	Tertile 2 (n = 651)	Tertile 3 (n = 647)	*P*
Age, years	47.4 ± 11.7	54.2 ± 10.8	51.9 ± 10.9	51.1 ± 9.9	< 0.001
Sex, male, n (%)	1271 (51.7)	432 (66.7)	401 (61.6)	353 (54.6)	< 0.001
Body mass index, kg/m^2^	23.2 ± 3.7	23.7 ± 3.6	23.2 ± 3.3	22.5 ± 3.2	< 0.001
Pretransplant dialysis vintage, months	0.4 (0, 2.4)	38.3 (23.1, 50.3)	83.1 (72.5, 92.8)	131.7 (114.9, 171.7)	< 0.001
**Primary renal disease, n (%)**					< 0.001
Diabetes mellitus	488 (22.5)	245 (37.8)	156 (24.0)	93 (14.4)	
Hypertension	279 (12.9)	110 (17.0)	129 (19.8)	137 (21.2)	
Glomerulonephritis	795 (36.6)	154 (23.8)	194 (29.8)	207 (32.0)	
Polycystic kidney disease	121 (5.6)	28 (4.3)	34 (5.2)	33 (5.1)	
Others	488 (22.5)	111 (17.1)	138 (21.2)	177 (27.4)	
Cold ischemic time, min	NA	295.9 ± 142.9	284.6 ± 132.9	286.1 ± 132.0	0.362
Desensitization, n (%)	729 (33.6)	10 (1.5)	5 (0.8)	14 (2.2)	< 0.001
**Comorbid conditions, n (%)**					
Hypertension	1947 (89.7)	609 (94.0)	598 (91.9)	554 (85.6)	< 0.001
Diabetes mellitus	630 (29.0)	300 (46.3)	191 (29.3)	116 (17.9)	< 0.001
Cardiovascular disease	128 (5.9)	104 (16.0)	100 (15.4)	106 (16.4)	< 0.001
HLA mismatch number	3.63 ± 1.74	3.37 ± 2.04	3.85 ± 1.73	3.83 ± 1.66	< 0.001
Donor age, years	46.7 ± 11.7	51.2 ± 15.1	49.1 ± 14.1	47.3 ± 13.3	< 0.001
Donor sex, male, n (%)	945 (43.5)	445 (68.7)	464 (71.3)	448 (69.4)	< 0.001
**Donor comorbid conditions, n (%)**					
Hypertension	210 (9.7)	195 (30.1)	160 (24.6)	136 (21.0)	< 0.001
Diabetes mellitus	22 (1.0)	94 (14.5)	67 (10.3)	70 (10.8)	< 0.001
**Donor cause of brain death, n (%)**					0.511
Head trauma	NA	206 (31.8)	209 (32.1)	221 (34.2)	
CVA	NA	232 (35.8)	215 (33.0)	222 (34.3)	
Hypoxic brain injury	NA	168 (25.9)	193 (29.6)	174 (26.9)	
Others	NA	42 (6.5)	34 (5.2)	30 (4.6)	
Donor CRRT, n (%)	0	36 (5.6)	45 (6.9)	45 (7.0)	0.054
**Induction immunosuppression, n (%)**					
Anti-thymocyte globulin	316 (14.6)	192 (29.6)	179 (27.5)	213 (33.0)	< 0.001
Basiliximab	1875 (86.4)	470 (72.5)	476 (73.1)	464 (71.9)	< 0.001
**Immunosuppressants, n (%)**					
Tacrolimus	2081 (95.9)	639 (98.6)	639 (98.2)	636 (98.6)	< 0.001
Mycophenolate	2021 (93.1)	606 (93.5)	605 (92.9)	596 (92.4)	0.405
Corticosteroid	2139 (98.5)	638 (98.5)	637 (97.9)	632 (98.0)	0.272

*LDKT* living donor kidney transplant, *HLA* human leukocyte antigen, *CVA* cerebrovascular accident, *CRRT* continuous renal replacement therapy.

### Primary composite outcome of both patient death and graft failure

**Figure 2:**
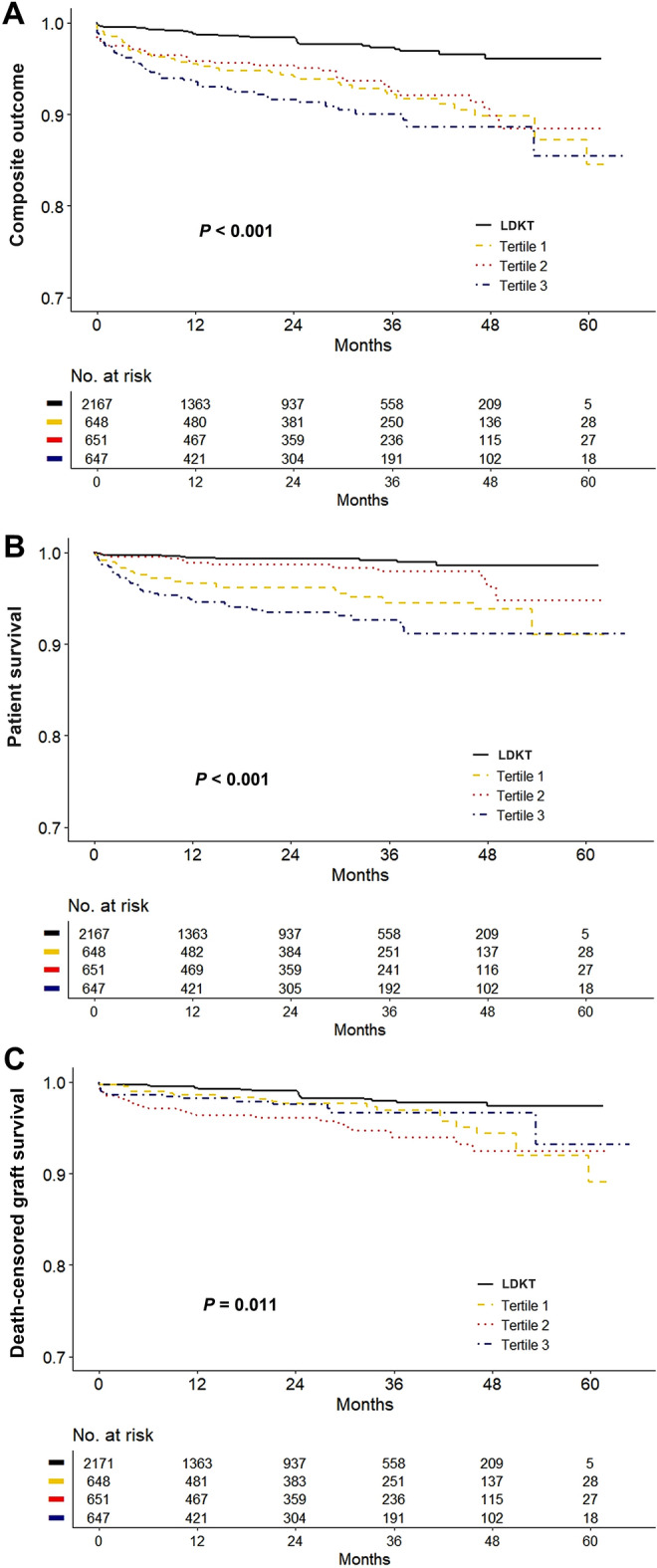
Kaplan–Meier curves for outcomes. (**A**) Composite outcome. (**B**) Patient survival. (**C**) Death-censored graft survival.

A total of 142 composite events (7.3%) were observed in DDKT recipients (Table 2). In the Kaplan–Meier curve for the composite outcome, all tertile groups showed lower event-free survival compared with the LDKT group (log-rank *P* < 0.001) (Fig. 2A). In addition, the results of the multivariable Cox regression models for composite outcome showed a consistently higher risk in increasing tertile order (model 3; first tertile: adjusted hazard ratio [aHR] 3.24, 95% confidence interval [CI] 1.87–5.62, *P* < 0.001; second tertile: aHR 3.25, 95% CI 1.85–5.73, *P* < 0.001; third tertile: aHR 5.31, 95% CI 3.12–9.03, *P* < 0.001) (Table 3). Among the DDKT recipients, third tertile group independently showed increased risk of composite outcome and second tertile group showed non-inferior results compared with first tertile group (Supplementary Table 1). A longer pretransplant dialysis vintage in DDKT was also consistently associated with an increased risk of composite outcome compared with LDKT in subgroups defined by age, sex, body mass index (BMI), Biopsy-proven acute rejection (BPAR), donor age, and comorbid diabetes (Supplementary Fig. 1).

**Table 2:** Outcomes after kidney transplant.

	LDKT	Tertile 1	Tertile 2	Tertile 3	*P*
**Patient death, n (%)**	14 (0.6)	28 (4.3)	12 (1.8)	37 (5.7)	< 0.001
Cardiovascular death, n (%)	0 (0.0)	5 (0.8)	2 (0.3)	4 (0.6)	0.002
Infection-related death, n (%)	0 (0.0)	12 (1.9)	5 (0.8)	16 (2.5)	< 0.001
Other causes of death, n (%)	14 (0.6)	11 (1.7)	5 (0.8)	17 (2.7)	< 0.001
Graft failure, n (%)	27 (1.2)	20 (3.1)	30 (4.6)	15 (2.3)	< 0.001
BPAR, n (%)	237 (10.9)	75 (11.6)	78 (12.0)	73 (11.3)	0.883
Delayed graft function, n (%)	27 (1.2)	20 (3.1)	30 (4.6)	15 (2.3)	< 0.001

*LDKT* living donor kidney transplant, *BPAR* biopsy-proven acute rejection.

**Table 3:** Cox regression analysis of composite outcomes.

	Univariate		Model 1		Model 2		Model 3	
	HR (95% CI)	*P*	aHR (95% CI)	*P*	aHR (95% CI)	*P*	aHR (95% CI)	*P*
**Vintage group**								
LDKT	Reference		Reference		Reference		Reference	
Tertile 1	3.14 (2.04–4.84)	< 0.001	2.58 (1.66–4.01)	< 0.001	3.21 (1.88–5.49)	< 0.001	3.24 (1.87–5.62)	< 0.001
Tertile 2	2.94 (1.89–4.57)	< 0.001	2.66 (1.71–4.16)	< 0.001	3.29 (1.89–5.72)	< 0.001	3.25 (1.85–5.73)	< 0.001
Tertile 3	4.05 (2.66–6.16)	< 0.001	3.89 (2.54–5.95)	< 0.001	5.18 (3.07–8.74)	< 0.001	5.31 (3.12–9.03)	< 0.001
Age	1.04 (1.02–1.05)	< 0.001	1.03 (1.01–1.04)	< 0.001	1.01 (1.00–1.03)	0.116	1.01 (0.99–1.03)	0.193
Sex (ref: male)	0.69 (0.50–0.95)	0.022	0.76 (0.55–1.05)	0.094	0.86 (0.60–1.21)	0.379	0.84 (0.59–1.20)	0.341
BMI	1.06 (1.02–1.10)	0.006	1.06 (1.01–1.11)	0.009	1.05 (1.01–1.10)	0.024	1.06 (1.01–1.10)	0.022
Desensitization	0.75 (0.48–1.16)	0.189			2.00 (1.13–3.54)	0.017	0.98 (1.12–3.50)	0.019
Hypertension	1.73 (0.91–3.27)	0.094			1.62 (0.75–3.50)	0.221	1.60 (0.74–3.47)	0.234
Diabetes	2.03 (1.51–2.74)	< 0.001			1.59 (1.12–2.26)	< 0.001	1.62 (1.14–2.30)	0.008
Cardiovascular disease	3.19 (2.28–4.47)	< 0.001			1.92 (1.30–2.82)	0.001	1.88 (1.27–2.78)	0.002
ATG induction	1.44 (0.99–2.08)	0.056			1.22 (0.83–1.80)	0.310	1.25 (0.85–1.84)	0.265
Tacrolimus use	0.61 (0.31–1.20)	0.155			0.58 (0.29–1.14)	0.114	0.55 (0.28–1.09)	0.088
Donor’s age	1.02 (1.01–1.03)	0.004					1.01 (1.00–1.02)	0.162
Donor’s hypertension	1.55 (1.08–2.21)	0.017					0.85 (0.55–1.30)	0.447
Donor’s diabetes	1.90 (1.18–3.07)	0.008					0.90 (0.51–1.59)	0.711
HLA mismatches	1.09 (1.00–1.19)	0.059					1.04 (0.94–1.14)	0.437

*LDKT* living donor kidney transplant, *HR* hazard ratio, *CI* confidence interval, *aHR* adjusted hazard ratio, *BMI* body mass index, *ATG* anti-thymocyte globulin, *HLA* human leukocyte antigen.

### Patient death

Overall, 77 deaths (3.9%) among DDKT recipients occurred within the study period (Table 4). Eleven patients died from cardiovascular disease (14.3% of total deaths), and 33 died from infectious causes (42.9% of total deaths). The Kaplan–Meier curve showed that patient survival in DDKT was significantly lower than in LDKT (*P* < 0.001) (Fig. 2B). After accounting for confounding factors, the rate of patient death was significantly higher in all DDKT groups compared with the LDKT group (model 3; first tertile: aHR 5.29, 95% CI 2.30–12.16, *P* < 0.001; second tertile: aHR 3.10, 95% CI 1.21–7.99, *P* = 0.019; third tertile: aHR 11.12, 95% CI 4.94–25.00, *P* < 0.001). Older age, pretransplant desensitization, comorbid diabetes, and cardiac disease were independent risk factors for mortality in the multivariable Cox regression model (all *P* < 0.05). Among the DDKT recipients, only third tertile group independently showed increased mortality compared with first tertile group (Supplementary Table 2).

**Table 4:** Cox regression analysis of patient death.

	Univariate		Model 1		Model 2		Model 3	
	HR (95% CI)	*P*	aHR (95% CI)	*P*	aHR (95% CI)	*P*	aHR (95% CI)	*P*
**Vintage group**								
LDKT	Reference		Reference		Reference		Reference	
Tertile 1	5.62 (2.95–10.69)	< 0.001	3.63 (1.89–6.97)	< 0.001	5.27 (2.33–11.6)	< 0.001	5.29 (2.30–12.16)	< 0.001
Tertile 2	2.48 (1.15–5.37)	0.021	1.94 (0.89–4.22)	0.094	3.06 (1.20–7.81)	0.019	3.10 (1.21–7.99)	0.019
Tertile 3	8.39 (4.54–15.54)	< 0.001	7.55 (4.05–14.08)	< 0.001	11.35 (5.11–25.20)	< 0.001	11.12 (4.94–25.00)	< 0.001
Age	1.08 (1.06–1.11)	< 0.001	1.07 (1.05–1.10)	< 0.001	1.05 (1.02–1.08)	< 0.001	1.05 (1.03–1.08)	< 0.001
Sex (ref: male)	0.52 (0.32–0.82)	0.005	0.56 (0.35–0.90)	0.017	0.66 (0.40–1.09)	0.105	0.63 (0.37–1.05)	0.076
BMI	1.05 (1.00–1.11)	0.064	1.06 (0.99–1.13)	0.081	1.04 (0.97–1.11)	0.299	1.03 (0.96–1.10)	0.389
Desensitization	0.66 (0.35–1.24)	0.198			2.62 (1.10–6.24)	0.029	2.63 (1.11–6.27)	0.029
Hypertension	1.82 (0.74–4.48)	0.194			1.50 (0.46–4.86)	0.497	1.44 (0.45–4.67)	0.541
Diabetes	3.01 (1.99–4.56)	< 0.001			1.88 (1.17–3.03)	0.009	1.83 (1.14–2.96)	0.013
Cardiovascular disease	5.35 (4.50–8.20)	< 0.001			2.44 (1.51–3.95)	< 0.001	2.44 (1.50–3.96)	< 0.001
ATG induction	1.72 (1.06–2.79)	0.029			1.38 (0.84–2.29)	0.206	1.39 (0.84–2.31)	0.199
Tacrolimus use	0.48 (0.21–1.11)	0.086			0.46 (0.20–1.06)	0.068	0.44 (0.19–1.02)	0.055
Donor’s age	1.02 (1.00–1.04)	0.021					1.00 (0.98–1.02)	0.993
Donor’s hypertension	1.75 (1.09–2.82)	0.021					0.76 (0.43–1.35)	0.351
Donor’s diabetes	2.83 (1.60–5.02)	< 0.001					1.28 (0.65–2.49)	0.475
HLA mismatches	1.12 (0.99–1.27)	0.081					1.05 (0.92–1.20)	0.496

*LDKT* living donor kidney transplant, *HR* hazard ratio, *CI* confidence interval, *aHR* adjusted hazard ratio, *BMI* body mass index, *ATG* anti-thymocyte globulin, *HLA* human leukocyte antigen.

### Graft failure

**Figure 3:**
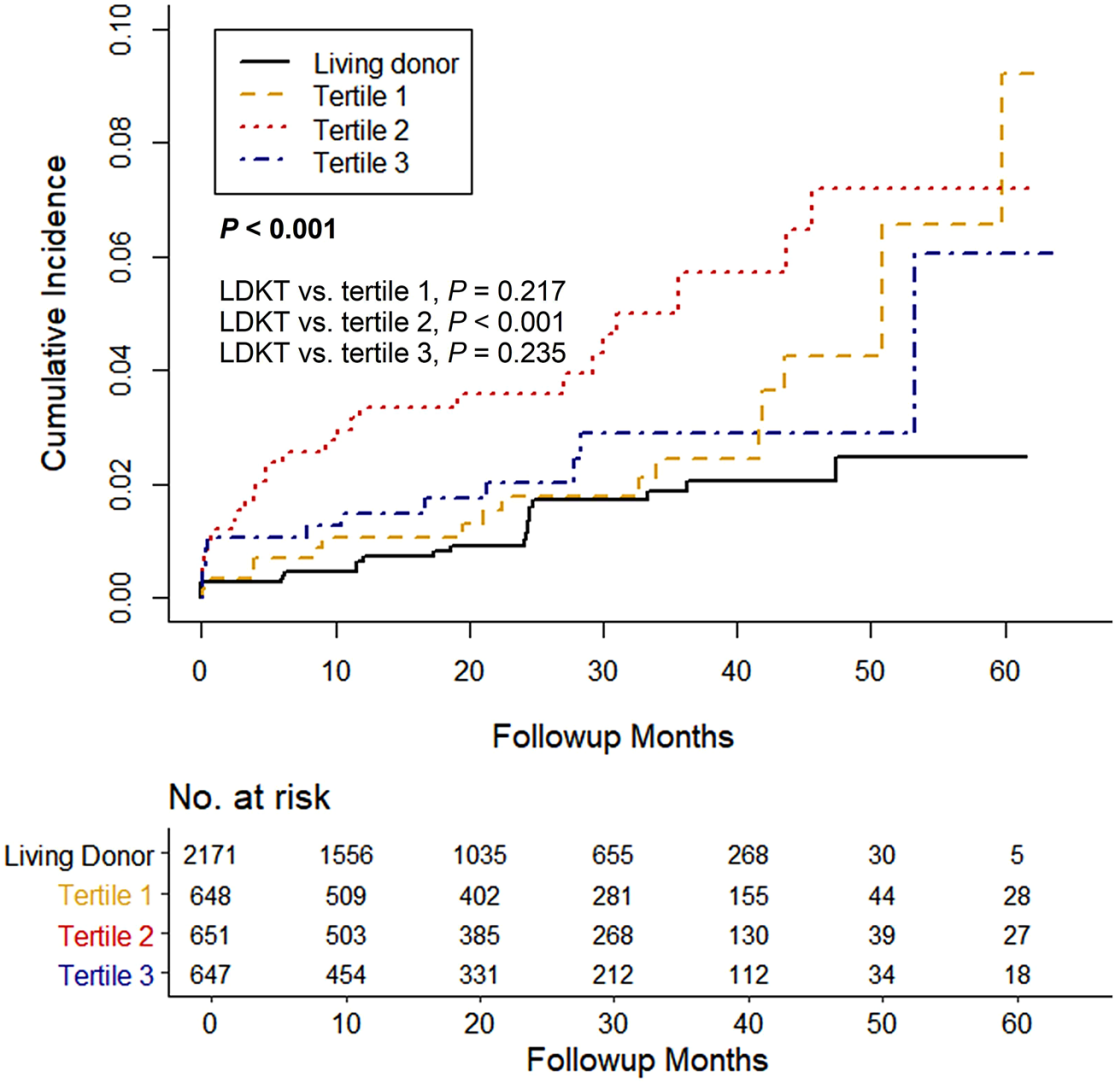
Cumulative incidence curves using the competing risk analysis method for graft failure stratified by duration of pretransplant dialysis. The Aalen–Johansen method was used for incidence estimate, and patient death was considered as a competing risk. *LDKT* living donor kidney transplant.

**Figure 4:**
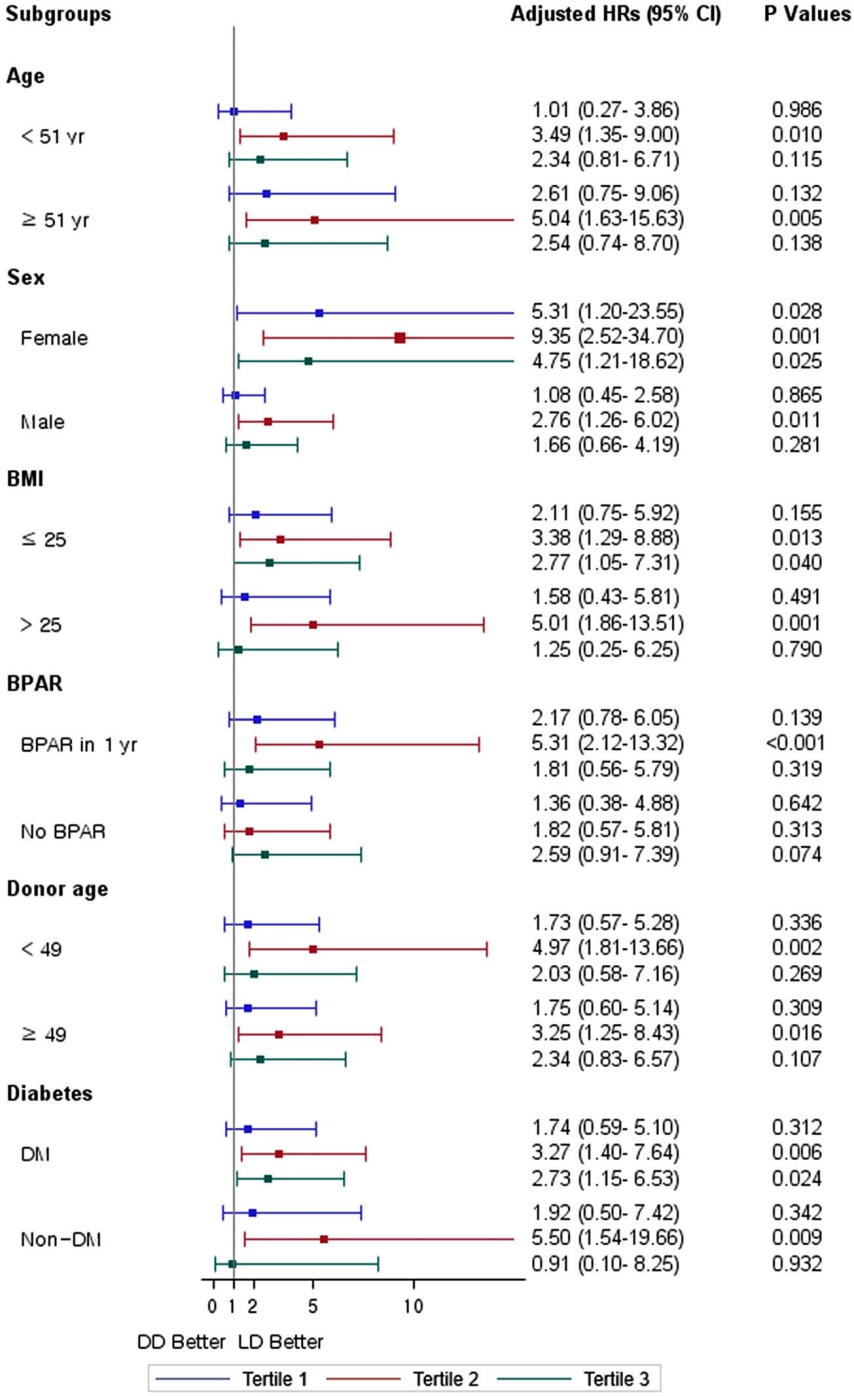
Subgroup competing risk analysis for graft failure. *LDKT* living donor kidney transplant, *BMI* body mass index, *BPAR* biopsy-proven acute rejection, *DM* diabetes mellitus, *HR* hazard ratio, *CI* confidence interval.

Sixty-five DDKT recipients (3.3%) lost graft function during the study period (Table 2). The Kaplan–Meier curve showed that death-censored graft survival was significantly lower in DDKT, with the lowest in the second tertile groups (log-rank *P* = 0.011) (Fig. 2C). For a more accurate estimation of graft failure, we applied a competing risk analysis model. The Aalen–Johansen method revealed a significantly increased cumulative incidence for graft failure in second tertile DDKT patients compared with LDKT patients (*P* < 0.001) (Fig. 3). The multivariable proportional hazards model for the subdistribution of the competing risk showed that the first tertile group consistently showed no increased risk of graft failure compared with the LDKT group. However, the second and third tertiles of pretransplant dialysis vintage were associated with a significantly higher risk of graft failure (model 3; second tertile: aHR 3.59, 95% CI 1.69–7.63, *P* < 0.001; third tertile: aHR 2.37, 95% CI 1.06–5.33, *P* = 0.037) (Table 5). Recipient BMI and donor age were independent risk factors of graft failure in DDKT recipients (both *P* < 0.05). In the subgroup competing risk analysis for graft failure, the first tertile group show no increased risk of graft failure in most subgroups compared with the LDKT group (Fig. 4). Among the DDKT recipients, there was no increase of graft failure risk in second and third tertile groups compared with first tertile group (Supplementary Table 3).

**Table 5:** Subdistribution hazard regression models of graft failure.

	Univariate		Model 1		Model 2		Model 3	
	HR (95% CI)	*P*	aHR (95% CI)	*P*	aHR (95% CI)	*P*	aHR (95% CI)	*P*
**Vintage group**								
LDKT	Reference		Reference		Reference		Reference	
Tertile 1	1.65 (0.89–3.06)	0.113	1.71 (0.92–3.18)	0.090	1.90 (0.86–4.20)	0.116	2.03 (0.88–4.66)	0.096
Tertile 2	3.04 (1.75–5.27)	< 0.001	3.19 (1.82–5.58)	< 0.001	3.55 (1.73–7.30)	< 0.001	3.59 (1.69–7.63)	< 0.001
Tertile 3	1.53 (0.78–3.00)	0.215	1.66 (0.84–3.27)	0.142	2.12 (0.96–4.67)	0.063	2.37 (1.06–5.33)	0.037
Age	1.00 (0.98–1.02)	0.805	0.99 (0.97–1.01)	0.354	0.99 (0.96–1.01)	0.188	0.98 (0.96–1.00)	0.085
Sex (ref: male)	0.93 (0.60–1.45)	0.744	1.02 (0.65–1.61)	0.925	1.12 (0.69–1.82)	0.643	1.16 (0.72–1.88)	0.547
BMI	1.06 (1.00–1.12)	0.046	1.06 (1.00–1.13)	0.042	1.08 (1.02–1.15)	0.015	1.09 (1.03–1.16)	0.006
Desensitization	0.86 (0.47–1.59)	0.637			1.66 (0.76–3.64)	0.204	1.63 (0.75–3.56)	0.222
Hypertension	1.62 (0.66–3.98)	0.294			1.68 (0.64–4.44)	0.296	1.74 (0.66–4.61)	0.263
Diabetes	1.27 (0.81–2.00)	0.304			1.29 (0.76–2.19)	0.354	1.36 (0.80–2.30)	0.258
Cardiovascular disease	1.42 (0.77–2.60)	0.262			1.17 (0.59–2.34)	0.650	1.08 (0.53–2.20)	0.840
ATG induction	1.13 (0.63–2.04)	0.680			0.97 (0.52–1.80)	0.922	1.01 (0.54–1.89)	0.979
Tacrolimus use	0.87 (0.27–2.74)	0.808			0.78 (0.24–2.48)	0.668	0.74 (0.23–2.38)	0.607
Donor’s age	1.02 (1.00–1.03)	0.068					1.02 (1.00–1.04)	0.040
Donor’s hypertension	1.32 (0.77–2.27)	0.308					0.96 (0.49–1.89)	0.903
Donor’s diabetes	0.96 (0.39–2.36)	0.935					0.42 (0.12–1.45)	0.169
HLA mismatches	1.04 (0.92–1.17)	0.553					1.02 (0.90–1.16)	0.721

*LDKT* living donor kidney transplant, *HR* hazard ratio, *CI* confidence interval, *aHR* adjusted hazard ratio, *BMI* body mass index, *ATG* anti-thymocyte globulin, *HLA* human leukocyte antigen.

Supplementary Fig. 2 shows the serial changes in tacrolimus trough level across the groups. Overall, the mean tacrolimus trough levels were similar after transplant, and the level was significantly higher 4 years after KT in the second and third tertiles compared with the LDKT group.

### Biopsy-proven acute rejection

BPAR incidence did not differ among LDKT and DDKT groups during the study period (Table 2). The Kaplan–Meier curve showed BPAR-free survival within 1 and 5 years after transplant also did not vary among the groups (Supplementary Fig. 3). In the univariable and multivariable Cox regression models for BPAR, pretransplant dialysis vintage in DDKT was not associated with the risk of BPAR compared with the LDKT (all *P* > 0.05) (Supplementary Table 4).

## Discussion

This study demonstrated that a longer pretransplant dialysis is associated with a worse prognosis in DDKT. The risk of the composite outcome of both patient death and graft failure, indicating actual graft failure, was increased with the pretransplant dialysis vintage. Considering patient death as a competing risk, the risk of graft failure in DDKT recipients with a shorter pretransplant dialysis duration was not inferior to that of LDKT recipients. This finding supports the need for efforts to shorten the waiting time for DDKT.

A recent Austrian national cohort study confirmed that pretransplant dialysis duration did not affect the risk of graft failure in transplants performed since 2000^13^. They suggested that erythropoiesis-stimulating agents have decreased transfusion frequency and sensitization risk, thereby improving transplant outcomes. However, the patients included in the Austrian study had undergone a relatively shorter period of pretransplant dialysis than in the present study; thus, the effect of pretransplant dialysis vintage could not be clearly identified in this study. Korean patients must start dialysis before registering on the waiting list for DDKT, and the waiting period until DDKT was much longer than in other countries. Therefore, our cohort data were enabled the analysis of the effect of long-term pretransplant dialysis on transplant outcomes, and long pretransplant dialysis resulted in decreased graft survival. This is consistent with previous studies reporting that a longer pretransplant dialysis increased the risk of sensitization and was associated with the increased risk of graft failure^9,16^.

For accurate analysis, we applied several competing risk analysis methods to estimate the association of pretransplant dialysis vintage with the risk of graft failure. Traditional analysis methods, such as Kaplan–Meier method and Cox regression model, for kidney allograft survival are limited in estimating long-term graft survival^20^. The competing risk analysis provides more accurate estimates of graft survival by correcting for the erroneous assumption that dead recipients remain at risk for graft failure^11^. We subsequently confirmed that DDKT recipients undergoing pretransplant dialysis up to 60.5 months did not have an increased risk of graft failure compared with LDKT recipients^8^. Previously, Meier-Kriesche et al.^16^ reported that the waiting time on dialysis was the strongest modifiable risk factor for kidney transplant outcome, and the effect was prevalent in DDKT. They found the effect of pretransplant dialysis on graft survival was equivalent between DDKT recipients undergoing pretransplant dialysis up to 6 months and LDKT recipients undergoing pretransplant dialysis up to 2 years. Our study showed DDKT recipients with a longer period of dialysis had comparable allograft outcomes with LDKT patients. The differences in the pretransplant dialysis duration might be attributable to the improvement of the dialysis technique and the decrease of the impact of dialysis in DDKT. Furthermore, we used the Aalen–Johansen estimates of incidence, which is considered a more appropriate method of controlling the competing risk in long-term graft survival after KT than the Fine–Gray competing risk model or the traditional survival analysis^20^.

Our cohort data revealed that infection-related death was the most common cause of patient death in DDKT. Infection-related mortality and all-cause mortality were highest in KT recipients with extended pretransplant dialysis. One possible explanation could be the immunosuppression status. Recent advances in immunosuppressive agents have made KT possible in sensitized patients with extended dialysis, and strong immunosuppressive therapy is needed to prevent rejection in patients^13,21^. As Lenihan et al.^22^ reported the changing complications trend in KT recipients in the United States, the incidence of infectious disease remained similar over 10 years despite the improvements in overall transplant outcomes and serious cardiovascular events. A longer pretransplant dialysis duration was a risk factor for infection-related mortality in DDKT recipients^23,24^. We confirmed that a similar or higher tacrolimus concentration was prescribed up to 5 years in DDKT patients at high risk of infection. Therefore, for KT recipients who underwent an extended dialysis period, the intensity of immunosuppression needs to be individually tailored and modulated to consider the risk of serious infectious complications. This suggestion can be supported by the similarity in BPAR frequency among DDKT patients regardless of pretransplant dialysis duration.

This study features several strengths. We conducted the study using data on patients who underwent KT in recent years, during which advanced immunosuppression protocols such as a monitoring of donor-specific antibodies were applied to all patients, and pretransplant dialysis was performed using the latest methods. The results of previous studies might be limited in this respect because they included the patients who received transplants several decades ago^4,11,13,25^. In addition, we evaluated the effect of dialysis duration only in the DDKT recipients except for LDKT because DDKT often has more vulnerable recipients or allograft status than LDKT. Finally, this is the first study to evaluate the effect of pretransplant vintage on the clinical outcomes of DDKT in Asian cohorts. Differences were noted in the prevalence of ESKD, distribution of dialysis modality, healthcare resources, health insurance system, and ethnic characteristics between Western and Asian countries, which affect transplant outcomes^13^.

This study has some limitations. First, this was an observational study and inevitably has unmeasured confounding factors. However, we tried to reduce the residual confounders by adjusting for multivariable factors and using competing risk analysis methods. Second, the transplant centers varied in clinical practice quality. The quality of kidney transplant centers has a significant association with mortality^26^. However, the Data from the Korean Organ Transplant Registry (KOTRY) database included most of the major transplant centers in Korea, minimizing the center effect.

In conclusion, pretransplant dialysis duration is an independent risk factor for patient death and graft failure in DDKT recipients. DDKT recipients with shortened pretransplant dialysis periods had good graft survival comparable with that of LDKT recipients. Therefore, efforts to reduce the waiting time for DDKT are needed, such as timely enrollment to the waiting list and maintaining healthy recipient conditions. A national policy that emphasizes the pretransplant dialysis period on allocation should be prioritized to improve outcomes in DDKT.

## Methods

### Study design and patient population

Data from the KOTRY were used in this study. The KOTRY has prospectively collected Korean transplant data from nationwide 59 transplant centers since 2014^27^. All first-time single-organ LDKT recipients and DDKT recipients between 2014 and 2019 in the KOTRY database were included. Recipients younger than 19 years or those who underwent simultaneous multiorgan transplantation were excluded from the KOTRY. The detailed design and methods for the KOTRY are presented in a previously published article^28^. We divided DDKT recipients into three groups according to the pretransplant dialysis period then compared their clinical outcomes with those of the LDKT recipients who underwent pretransplant dialysis less than 6 months. The control group, LDKT recipients with pretransplant dialysis less than 6 months, showed the best graft survival in our previous study^8^.

### Data collection

A total of 6,118 newly kidney transplanted patients were registered in the KOTRY database during the study period. Among them, 1,529 LDKT recipients with pretransplant dialysis longer than 6 months and 472 recipients of second KT were excluded. The remaining 4,117 KT recipients were included in the analysis. The baseline demographic characteristics of the recipients and donors, laboratory data, graft failure, patient death, and occurrence of delayed graft function and rejection were obtained. Estimated glomerular filtration rate was calculated using the Modification of Diet in Renal Disease Equation^29^.

### Outcomes

The main outcomes were graft failure, all-cause mortality, and the composite of both. Graft survival time was defined as the time from KT to the initiation of permanent dialysis, second KT, or end of follow-up. Patient survival time was defined as KT to death from any cause or end of follow-up. A competing risk analysis was applied to avoid censoring for patient death in analyzing the risk of graft failure. BPAR was also compared and diagnosed based on the Banff 07 classification^30^.

### Statistical analysis

Continuous variables are presented as mean ± standard deviation or median (IQR) depending on their distribution, and categorical variables are presented as number and percentage. One-way analysis of variance or the Kruskal–Wallis test was used to determine the differences in continuous variables, as appropriate, whereas for categorical variables, Pearson’s chi-square test or Fisher’s exact test was used. The Cochran–Armitage trend test was performed to analyze the time trend of pretransplant dialysis vintage. Kaplan–Meier curves and the log-rank test were used to compare the differences in graft survival, patient survival, composite event-free survival, and early BPAR-free survival among the KT groups. The competing risk method using the Aalen–Johansen estimate was used to compare the cumulative incidence rates of graft failure among the groups^31,32^, and patient death was set as a competing event. The association between pretransplant dialysis vintage and clinical outcomes, including composite outcomes, was further determined using multivariable Cox proportional hazard regression models. The adjustment factors selected were baseline characteristics and clinically relevant variables. Patient death can be a competing event on graft failure; thus, we used the Fine and Gray competing risk model for subdividing a competing risk (patient death) to compare the risk of graft failure^33^. Subgroup analyses by age, sex, body mass index, early BPAR, donor age, and comorbid diabetes were performed for patient death and graft failure. The graft failure was also analyzed by competing risk analysis in subgroup analysis. Statistical analyses were performed with SPSS version 22.0 (IBM Corp., Armonk, NY, USA) and R (R Foundation for Statistical Computing, Vienna, Austria; www.r-project.org). *P* values less than 0.05 were considered statistically significant.

### Ethics declarations

The data do not contain personal information and do not infringe on the privacy of patients. This study was approved by the Institutional Review Board of the Kyungpook National University Hospital (2020-11-057). All patients provided written informed consent before participation, and the study was conducted according to the tenets of the 2013 Declaration of Helsinki and the Declaration of Istanbul 2008.

## Supplementary Information

Supplementary Information.

## Data Availability

The datasets generated during and/or analysed during the current study are available from the corresponding author on reasonable request.
