# Supplementary material for: Effect of pretransplant dialysis vintage on clinical outcomes in deceased donor kidney transplant
Source: Sci Rep. 2022 Oct 21;12:17614. doi: 10.1038/s41598-022-20003-2 (PMC9587225; doi:10.1038/s41598-022-20003-2)
Supplement: Supplementary file 1 — Supplementary Information. [file 41598_2022_20003_MOESM1_ESM.pdf]

Effect of pretransplant dialysis vintage on clinical outcomes in deceased donor kidney transplant

Jeong-Hoon Lim[1], Yena Jeon[2], Deok Gie Kim[3], Yeong Hoon Kim[4], Joong Kyung Kim[5], Jaeseok Yang[6], Myoung Soo Kim[7], Hee-Yeon Jung[1], Ji-Young Choi[1], Sun-Hee Park[1], Chan-Duck Kim[1], Yong-Lim Kim[1], and Jang-Hee Cho[1], the Korean Organ Transplantation Registry Study Group

[1]Department of Internal Medicine, School of Medicine, Kyungpook National University, Daegu, South Korea

[2]Department of Statistics, Kyungpook National University, Daegu, South Korea

[3]Department of Surgery, Yonsei University Wonju College of Medicine, Wonju Severance Christian Hospital, Wonju, South Korea

[4]Department of Internal Medicine, Inje University Busan Paik Hospital, Busan, South Korea

[5]Department of Internal Medicine, Bongseng Memorial Hospital, Busan, South Korea

[6]Department of Internal Medicine, Yonsei University College of Medicine, Seoul, South Korea

[7]Department of Surgery, Yonsei University College of Medicine, Seoul, South Korea

Correspondence information:

Jang-Hee Cho, MD, PhD

Professor

Division of Nephrology, Department of Internal Medicine

Kyungpook National University Hospital, 130 Dongdeok-ro, Jung-gu, Daegu, 41944, South Korea

Tel: +82-53-200-5550

Fax: +82-53-426-2046

Email: jh-cho@knu.ac.kr

Supplementary Information Contents

Supplementary Table 1. Cox regression analysis of composite outcomes among DDKT tertile groups

Supplementary Table 2. Cox regression analysis of patient death among DDKT tertile groups

Supplementary Table 3. Subdistribution hazard regression models of graft failure among DDKT tertile groups

Supplementary Table 4. Cox regression analysis of biopsy-proven acute rejection

Supplementary Figure 1. Subgroup analysis for composite outcome.

Supplementary Figure 2. Serial changes in tacrolimus trough level.

Supplementary Figure 3. Kaplan–Meier curves for biopsy-proven acute rejection. (A) Biopsy-proven acute rejection during 12 months after transplant. (B) Biopsy-proven acute rejection during the observation period.

.

Supplementary Table 1. Cox regression analysis of composite outcomes among DDKT tertile groups

	Univariate		Model 1[a]		Model 2[b]		Model 3[c]	
	HR (95% CI)	*P*	aHR (95% CI)	*P*	aHR (95% CI)	*P*	aHR (95% CI)	*P*
Vintage group								
Tertile 1	Reference		Reference		Reference		Reference	
Tertile 2	0.87 (0.54–1.39)	0.558	0.98 (0.61–1.57)	0.918	1.06 (0.66–1.72)	0.805	1.04 (0.64–1.70)	0.875
Tertile 3	1.24 (0.80–1.92)	0.340	1.50 (0.96–2.36)	0.078	1.67 (1.05–2.65)	0.030	1.70 (1.06–2.73)	0.028

Abbreviations: DDKT, deceased donor kidney transplantation; HR, hazard ratio; CI, confidence interval; aHR, adjusted hazard ratio.

[a]Adjusted for age, sex, and body mass index.

[b]Adjusted for age, sex, body mass index, pretransplant desensitization, underlying comorbidities (diabetes, hypertension, and cardiac disease), ATG induction, and maintenance tacrolimus use.

[c]Adjusted for age, sex, body mass index, pretransplant desensitization, underlying comorbidities (diabetes, hypertension, and cardiac disease), ATG induction, maintenance tacrolimus use, donor’s age, donor’s hypertension and diabetes, and number of human leukocyte antigen mismatches.

Supplementary Table 2. Cox regression analysis of patient death among DDKT tertile groups

	Univariate		Model 1[a]		Model 2[b]		Model 3[c]	
	HR (95% CI)	*P*	aHR (95% CI)	*P*	aHR (95% CI)	*P*	aHR (95% CI)	*P*
Vintage group								
Tertile 1	Reference		Reference		Reference		Reference	
Tertile 2	0.45 (0.22–0.92)	0.029	0.54 (0.27–1.11)	0.095	0.60 (0.29–1.23)	0.162	0.61 (0.29–1.26)	0.177
Tertile 3	1.39 (0.82–2.36)	0.220	1.91 (1.10–3.31)	0.021	2.18 (1.24–3.84)	0.007	2.13 (1.19–3.79)	0.011

Abbreviations: DDKT, deceased donor kidney transplantation; HR, hazard ratio; CI, confidence interval; aHR, adjusted hazard ratio.

[a]Adjusted for age, sex, and body mass index.

[b]Adjusted for age, sex, body mass index, pretransplant desensitization, underlying comorbidities (diabetes, hypertension, and cardiac disease), ATG induction, and maintenance tacrolimus use.

[c]Adjusted for age, sex, body mass index, pretransplant desensitization, underlying comorbidities (diabetes, hypertension, and cardiac disease), ATG induction, maintenance tacrolimus use, donor’s age, donor’s hypertension and diabetes, and number of human leukocyte antigen mismatches.

Supplementary Table 3. Subdistribution hazard regression models of graft failure among DDKT tertile groups

	Univariate		Model 1[a]		Model 2[b]		Model 3[c]	
	HR (95% CI)	*P*	aHR (95% CI)	*P*	aHR (95% CI)	*P*	aHR (95% CI)	*P*
Vintage group								
Tertile 1	Reference		Reference		Reference		Reference	
Tertile 2	1.70 (0.86–3.37)	0.128	1.78 (0.90–3.52)	0.098	1.93 (0.96–3.90)	0.066	1.81 (0.89–3.70)	0.102
Tertile 3	0.96 (0.43–2.13)	0.922	1.04 (0.47–2.29)	0.925	1.11 (0.48–2.57)	0.817	1.18 (0.49–2.81)	0.716

Abbreviations: DDKT, deceased donor kidney transplantation; HR, hazard ratio; CI, confidence interval; aHR, adjusted hazard ratio.

[a]Adjusted for age, sex, and body mass index.

[b]Adjusted for age, sex, body mass index, pretransplant desensitization, underlying comorbidities (diabetes, hypertension, and cardiac disease), ATG induction, and maintenance tacrolimus use.

[c] Adjusted for age, sex, body mass index, pretransplant desensitization, underlying comorbidities (diabetes, hypertension, and cardiac disease), ATG induction, maintenance tacrolimus use, donor's age, donor's hypertension and diabetes, and number of human leukocyte antigen mismatches.

Supplementary Table 4. Cox regression analysis of biopsy-proven acute rejection

	Univariate		Model 1		Model 2		Model 3	
	HR (95% CI)	*P*	aHR (95% CI)	*P*	aHR (95% CI)	*P*	aHR (95% CI)	*P*
Vintage group								
Living Donor	Reference		Reference		Reference		Reference	
Tertile 1	0.92 (0.71–1.19)	0.502	0.96 (0.73–1.25)	0.743	0.98 (0.72–1.33)	0.888	0.96 (0.70–1.33)	0.811
Tertile 2	0.99 (0.77–1.28)	0.951	1.04 (0.80–1.34)	0.797	1.07 (0.79–1.44)	0.683	1.03 (0.75–1.41)	0.858
Tertile 3	0.99 (0.76–1.29)	0.961	1.05 (0.81–1.37)	0.707	1.06 (0.78–1.45)	0.705	1.09 (0.79–1.50)	0.596
Age	0.99 (0.98–1.00)	0.017	0.99 (0.98–1.00)	0.011	0.99 (0.98–1.00)	0.045	0.99 (0.98–1.00)	0.005
Sex (ref: male)	0.84 (0.69–1.01)	0.064	0.5 (0.70–1.03)	0.099	0.85 (0.69–1.04)	0.112	0.83 (0.68–1.02)	0.079
BMI	1.02 (1.00–1.05)	0.081	1.02 (1.00–1.05)	0.101	1.02 (1.00–1.05)	0.097	1.02 (1.00–1.05)	0.096

Desensitization	1.32 (1.06–1.65)	0.014	1.36 (1.05–1.76)	0.019	1.36 (1.05–1.75)	0.021
Hypertension	0.82 (0.61–1.09)	0.164	0.82 (0.60–1.12)	0.202	0.86 (0.63–1.18)	0.362
Diabetes	0.94 (0.77–1.16)	0.572	1.02 (0.81–1.28)	0.859	1.04 (0.83–1.31)	0.742
Cardiac	0.87 (0.63–1.19)	0.374	0.86 (0.60–1.23)	0.417	0.84 (0.58–1.20)	0.3347
ATG induction	1.52 (1.22–1.90)	<0.001	1.54 (1.22–1.94)	<0.001	1.57 (1.24–1.98)	<0.001
Tacrolimus use	1.03 (0.62–1.73)	0.9033	0.97 (0.58–1.63)	0.922	0.99 (0.58–1.69)	0.966
Donor’s age	1.02 (1.01–1.03)	<0.001			1.03 (1.02–1.03)	<0.001
Donor’s hypertension	1.09 (0.85–1.38)	0.498			0.94 (0.71–1.24)	0.645
Donor’s diabetes	0.79 (0.52–1.20)	0.262			0.69 (0.43–1.09)	0.114

HLA Mismatches	1.08 (1.02–1.14)	0.005	1.07 (1.01–1.13)	0.030

Abbreviations: HR, hazard ratio; CI, confidence interval; aHR, adjusted hazard ratio; BMI, body mass index; ATG, anti-thymocyte globulin; HLA, human leukocyte antigen.

Supplementary Figure 1. Subgroup analysis for composite outcome.

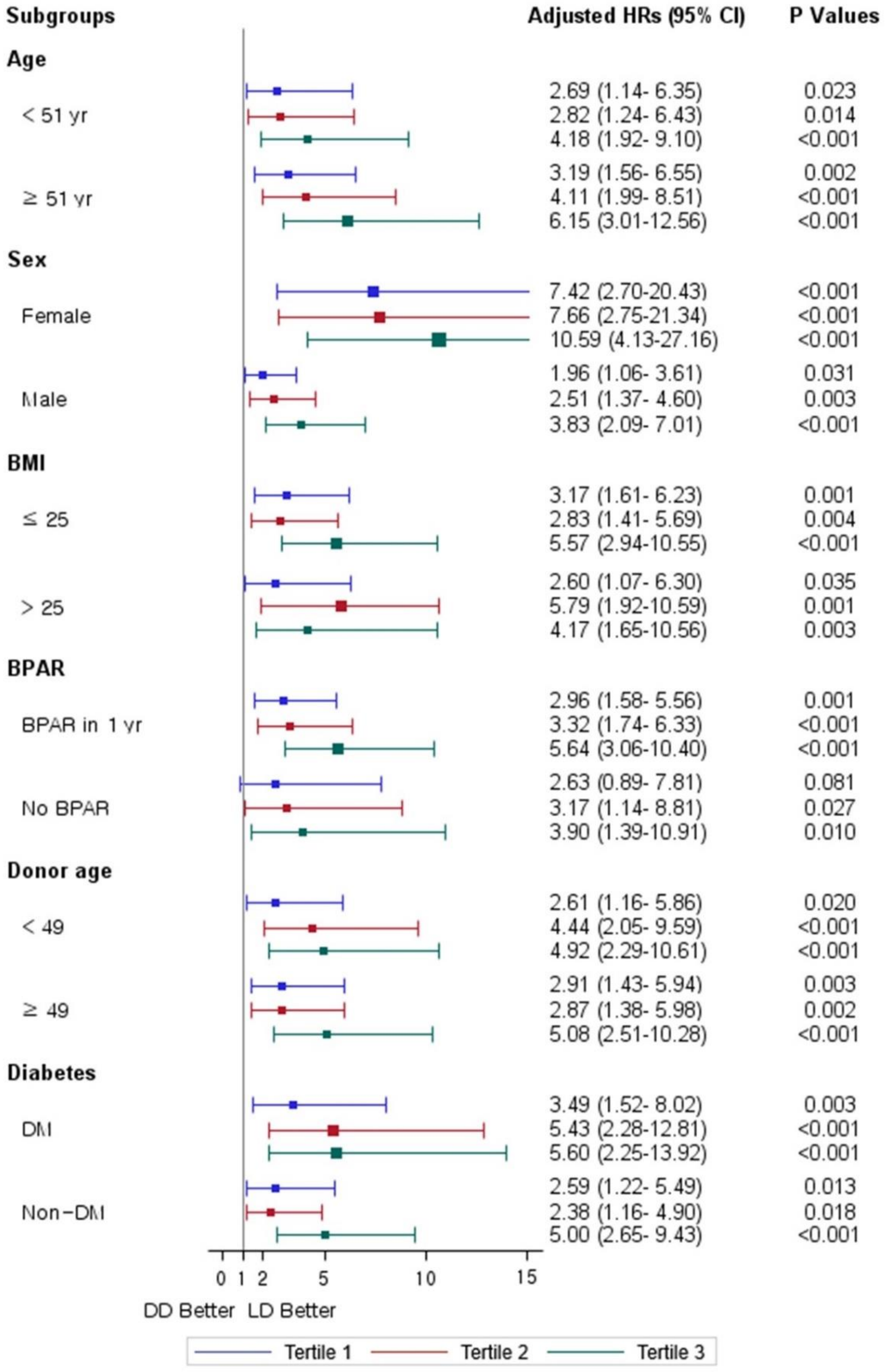

Abbreviations: LDKT, living donor kidney transplant; BMI, body mass index; BPAR, biopsy-proven acute rejection; DM, diabetes mellitus; HR, hazard ratio; CI, confidence interval.

Supplementary Figure 2. Serial changes in tacrolimus trough level.

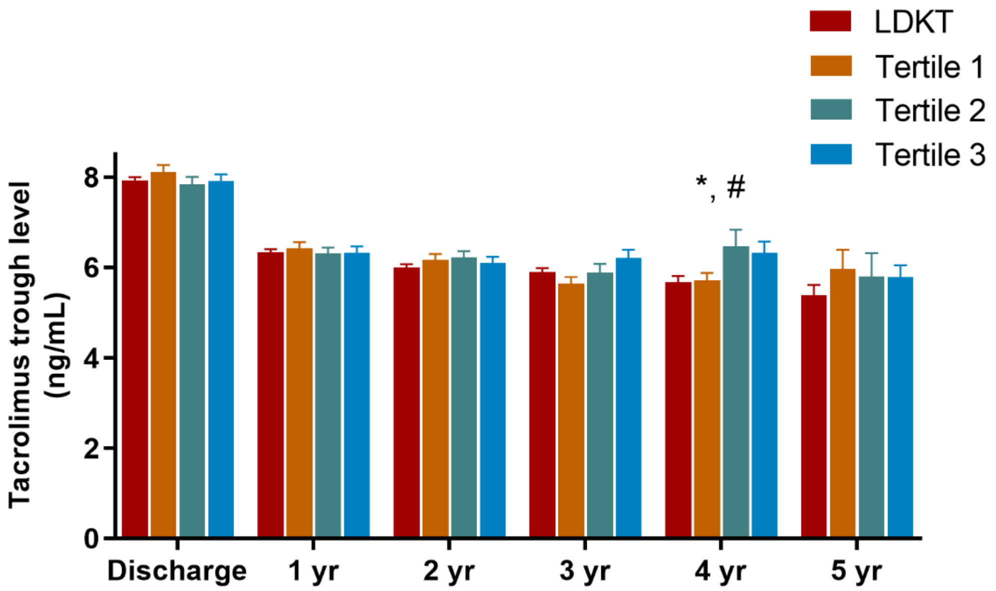

* indicates $P < 0.05$ between LDKT and second tertile; # indicates $P < 0.05$ between LDKT and third tertile.

Abbreviation: LDKT, living donor kidney transplant.

Supplementary Figure 3. Kaplan–Meier curves for biopsy-proven acute rejection. (A) Biopsy-proven acute rejection during 12 months after transplant. (B) Biopsy-proven acute rejection during the observation period.

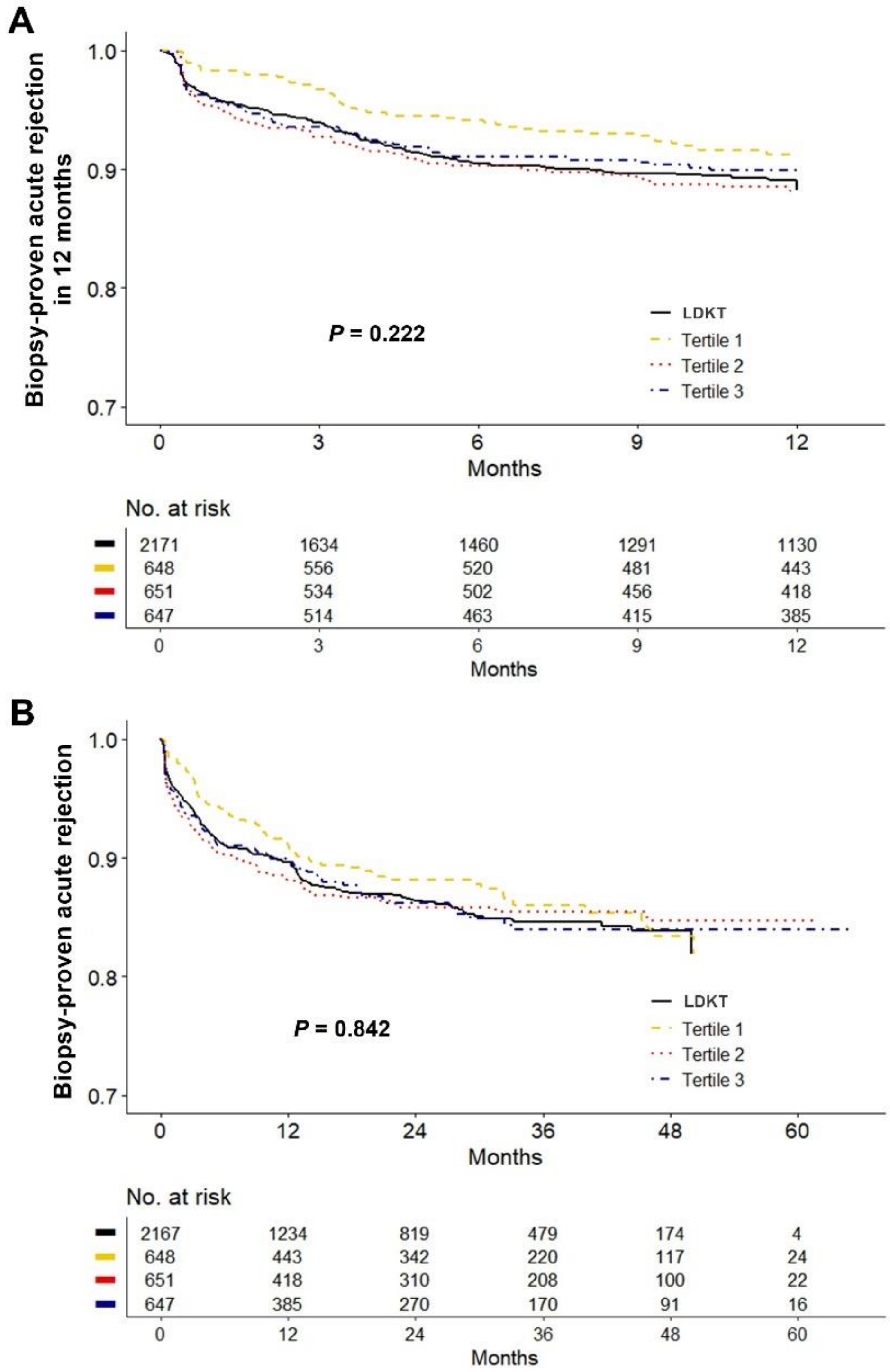

Abbreviation: LDKT, living donor kidney transplant.
